# Deviation Factors of Medical Operational Skills in Terms of Clinical Skills Training, Assessment Methods, and Digitalized Education — A Prisma-Based Review

**DOI:** 10.1007/s40670-025-02407-7

**Published:** 2025-07-19

**Authors:** Tianyi Liu, Marini Othman, Tin Tin Ting

**Affiliations:** 1 INTI International University, 71800 Negeri Sembilan, Malaysia; 2 Jiangsu Medical Vocational College, Yancheng, 224001 China; 3 Monash University Malaysia, Subang Jaya, Selangor Malaysia; 4 School of Information Technology, UNITAR International University, 47301 Petaling Jaya, Malaysia

**Keywords:** Operational skill, Clinical operational skill, Holistic framework, Objective Structured Clinical Examination, Health education system

## Abstract

The decline in clinical operational skills among recent medical graduates in China presents a pressing challenge, particularly in the context of the “Healthy China 2030” initiative. This study, which is a systematic review using the PRISMA method, systematically examines the contributing factors and potential solutions through a review of contemporary medical education methods, emphasizing the integration of traditional and digital approaches. This study aims to explore factors influencing operational skill acquisition, challenges in clinical training and assessment, and potential solutions identified in prior research, particularly through digital tools such as AI-assisted learning and virtual simulation. This study uses a theoretical framework centered on digitalized medical education, clinical skills training, and assessment methodologies, using content analysis to evaluate and generalize findings from peer-reviewed literature. The results indicate that while digital tools, such as simulation-based training and online platforms, improve student engagement and satisfaction, their impact on operational proficiency remains inconsistent. Traditional methods continue to dominate clinical training, underscoring the need for a blended approach that takes advantage of the strengths of both modalities. The findings also reveal systemic problems in China’s healthcare education system, including insufficient clinical rotation opportunities, lack of standardized assessment frameworks, and socioecological factors such as policy constraints, teacher-student ratios, and economic pressures. This study identifies significant gaps in existing research, particularly the absence of a unified framework to evaluate clinical skills and the limited focus on the digital competencies of undergraduate students. Additionally, demographic factors, including gender and socioeconomic status, emerge as critical influences on skill development. Further analysis suggests that future studies should prioritize longitudinal evaluations of digital education’s long-term impact on clinical practice, especially its application in actual clinical situations.

**Supplementary Information:**

The online version contains supplementary material available at 10.1007/s40670-025-02407-7.

## Introduction

Clinical operational skills refer to the psychomotor and cognitive abilities required for safe and effective patient care, encompassing procedures such as diagnostic reasoning, surgical techniques, and emergency management [49]. While this review has global implications, findings are contextualized within China’s healthcare education reforms under the “Healthy China 2030” initiative. National strategies emphasize healthcare modernization; however, medical education faces persistent challenges in aligning training with clinical demands. There are 15 major initiatives in it. Health education is one of the key initiatives. China has the largest population in the world. The Health Education Initiative was initiated to strengthen the health education system (Li et al., 2019). The integration of digital tools (e.g., simulations, virtual reality platforms) is critical for bridging gaps in medical education quality and accessibility [13]. The research aims to evaluate the current state of informatization in the health education system in China by comparing it with Western countries to understand the differences and determine the scope of improvement (Yan, 2020). Especially in the post-COVID-19 period, the increasing demand for skilled and experienced medical practitioners and the informatization of medical education in China have become critically important, this urgency necessitates a systematic review to identify gaps in current training methodologies and propose evidence-based solutions.

The status of teaching informatization in China has made progress in infrastructure, hardware, and software construction, while teachers’ perceptions, teaching methods, and modern educational technology have been greatly improved, and the construction of online courses has been effectively promoted. However, online teaching activities and the actual application of teaching need to be strengthened (Zhang et al., 2016). Although some private healthcare institutions have made some progress in the informatization of the education process by applying modern technologies in the skill development of practitioners, most of the public health system still lacks it (Ma et al., 2022). It follows the traditional model of healthcare education. There is evidence of declining medication practice abilities in students graduating from medical colleges in recent years (Luo et al., 2016), which is consistent with what the author has observed about medical graduates from Jiangsu Medical Vocational College. The country must reform its health infrastructure to compensate for the losses due to COVID-19. At the same time, there is an increasing demand for healthcare services for the growing population [22]. Therefore, it requires finding out the influential factors behind the decline in medical practice ability among recently graduated medical students, as well as using informatization to improve the quality of education of these students to increase their competency and efficiency.

The objective of this study is to explore relevant studies on digitalized medical education, clinical skills training, digital education, and assessment methods in medical education. The second objective of this study is to identify the influential factors (covariates) that impact clinical operational skills among Chinese medical graduates. A total of 408 records were initially identified, which were subsequently screened to exclude duplicates and irrelevant articles, producing 39 studies for a comprehensive review to answer the following research question:

- RQ1: What are the current studies in clinical skills training, assessment methods, and digitalized medical education?
- RQ2: How do the influential factors (covariates) impact clinical operational skills among Chinese medical graduates?

The content of this paper is organized into coherent sections covering methodology, results, discussion, and conclusions. The “Methodology” section outlines the PRISMA framework for a systematic literature search, detailing the inclusion criteria and thematic categorization of studies into areas such as clinical skills training, assessment methods, digitalized medical education, and social-ecological factors. The “Results” section highlights findings on key topics, including models and taxonomies for clinical skills, evaluation frameworks like Kirkpatrick’s levels and Miller’s pyramid, and the effectiveness of digitalized education modalities. It also examines social-ecological factors like policy, teacher-student ratios, and demographic influences on clinical performance. The “Discussion” identifies research gaps, such as the limited exploration of operational skills decline among interns, while the “Conclusions” summarize findings, highlight research deficiencies, and suggest directions for integrating digital and traditional education to advance medical training practices.

## Methodology

### PRISMA Methodology

This review of the literature conducted an extensive literature search using a systematic review method — PRISMA methodology. First, the researcher used keywords such as “digitalized medical education,” “clinical skills training,” and “assessment methods” to search the PubMed, Web of Science, and Google Scholar databases. Most of the literature was published between 2018 and 2023, and only three works were published in 1979 [6], 1986 [3], and 1993 [5]. When selecting the literature, the researcher used the following inclusion criteria: (1 related to digital education or training in clinical skills in medical education,(2 published in peer-reviewed journals; (3 provided empirical research data. Studies with an imprecise research design or a small sample size were excluded. (4 Only studies published in English or Chinese were included due to resource constraints. Subsequently, the researcher classified the selected literature according to keywords. To mitigate selection bias, two independent researchers screened titles and abstracts, with discrepancies resolved through consensus. When analyzing this literature, the research focused on comparing the assessment tools and methods used in different studies, evaluating their validity and reliability, and summarizing the main findings and limitations of various studies. This review adopted a content analysis approach to systematically summarize and generalize the main themes and research findings. Key data extracted included study design, sample size, intervention type (e.g., digital vs. traditional), outcome measures (e.g., OSCE scores), and limitations.

The following figure describes the systematic review process using searches of databases and registers. The flowchart tracks the progression of records through different phases: identification, screening, eligibility, and inclusion. Starting with 408 identified records, the process involved removing duplicates, automation-based exclusions, and irrelevant article types, which led to 368 records being screened. Further screening excluded 155 records, with 213 reports sought for retrieval. Of these, 201 reports were evaluated for eligibility, and exclusions were made for reasons such as being off-topic, not meeting inclusion criteria, or having unavailable data. Finally, 39 studies were included in the final review, demonstrating a rigorous and transparent approach to systematic analysis. Full search strings (e.g., “digital education AND clinical skills”) are provided in Supplementary File 1; this strategy ensures transparency, reproducibility, and alignment with PRISMA guidelines.

**Fig. 1:**
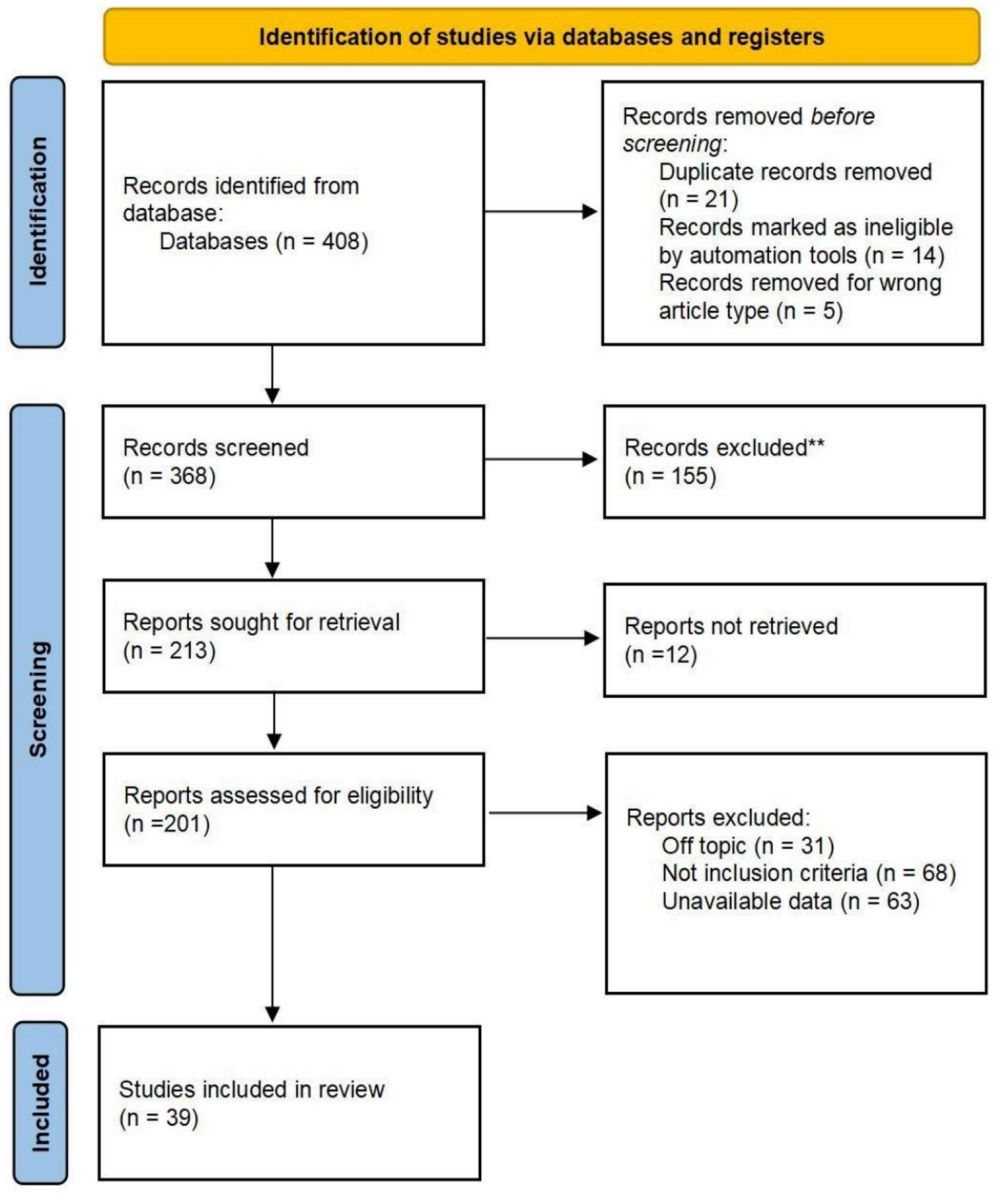
PRISMA flowchart

Figure 1 illustrates the selection process: 408 records were identified, 155 excluded due to irrelevance, and 39 studies retained for analysis. Key exclusions included off-topic studies (*n* = 31) and not inclusion criteria (*n* = 68).

### Result Categorization

The literature results in this study were classified according to several key themes, derived from the systematic review and content analysis of the selected literature. This categorization process was designed to provide a structured understanding of the main findings of the diverse studies analyzed. First, the literature was grouped according to the theoretical framework of this research, namely (1) clinical skills training, (2) assessment methods in medical education, (3) digitalized medical education, and (4) social-ecological factors. These categories were further divided into various subthemes: category 1: roles and skills of a medical doctor, and professional development; category 2: evaluation framework of medical education, and clinical assessments; category 3: digitalized medical education, modalities of digitalized medical education, digitalized approach versus traditional approach in medical education, and medical intern’s digital health competence.

This review of the literature first categorized the literature according to the roles and skills required of medical doctors (clinical skills training). Here, various studies presented different views to understand the professional development of medical students. For instance, Caiman [8] outlined multiple roles for doctors, and Boelen [5] proposed the “five-star doctor” model, which emphasizes healthcare provision, decision-making, communication, leadership, and resource management. These theoretical models were then linked to specific skills needed for clinical competence, as outlined by the World Federation for Medical Education (WFME, 2001) and the American Medical College Alliance (2010). This section underscored the multidimensional nature of medical education, from basic technical skills to interpersonal communication and leadership skills. In terms of professional development, the categorization highlighted several taxonomies that describe the progression of medical education. For example, [14] taxonomy of professional development provided a framework for categorizing levels of learning, including foundational knowledge, application, integration, and human dimension. This taxonomy helped categorize studies exploring the broader aspects of medical education, such as the role of the doctor in the provision and leadership. Furthermore, studies that focused on cognitive and psychomotor skills were classified as operational skill development, models such as the bidimensional skill development approach (Liu et al., 2017), which distinguishes between intellectual and operational competencies.

When examining clinical evaluations (assessment methods in medical education), the studies were classified according to the type of evaluation methods used. For example, [19] introduction of the Objective Structured Clinical Examination (OSCE) was classified into clinical skills assessments, along with other tools such as [23] training evaluation framework and Miller’s Pyramid, which assess various stages of clinical competence. These frameworks were used to evaluate the effectiveness of different assessment tools in measuring the complete capabilities of medical students, ranging from knowledge acquisition to the actual demonstration of clinical skills.

A significant portion of the literature was also categorized according to the integration of digitalized education methods into medical training (digitalized medical education). Studies focusing on digitalized medical education were grouped into various modalities, such as online education, mobile learning, and simulated learning. These categories were further divided based on the type of learning environment (online vs. online) and the impact of digital technologies, such as serious gaming and digital simulation, on the assessment of clinical skills. Research comparing digital and traditional educational approaches, especially in terms of learning outcomes and student satisfaction, was grouped into studies that examined the effectiveness and limitations of digital education in clinical training.

Finally, the categorization of studies on the impact of social-ecological factors, such as policy, teacher-student ratios, and individual student differences (e.g., gender and economic factors), was incorporated to highlight the broader contextual factors influencing clinical performance. These factors were classified under the theme “social and environmental influences” which explored how external elements affect the ability of medical students to develop and demonstrate their clinical skills. Overall, this categorization allowed a complete understanding of the current state of digitalized medical education and clinical skills training in medical education, identifying strengths and gaps in existing research.

## Systematic Literature Review Results

### Clinical Skills Training

#### Roles and Skills of a Medical Doctor

Operational skills are defined as the mode of activity composed of a series of body movements executed in a reasonable and perfect procedure [49], often referred to as motor skills. Caiman [8] identified multiple roles for doctors, including advocate, educator, reform spokesman, citizen, and leader. Boelen [5] proposed a concept of the “five-star doctor” who is adept at providing health care, making health decision-making, effective communication, leadership of community health, and management of health resources.

The World Federation for Medical Education [45] outlined global standards for medical graduates, emphasizing the need for graduates to handle patients’ health problems sympathetically, apply basic healthcare knowledge in practice, possess essential interpersonal communication skills, evaluate and apply new scientific insights, guide interactions among healthcare professionals, conduct preliminary research, exhibit professional qualities, advocate for patients’ interests, understand public health and health policy, and possess the ability to understand healthcare systems.

Zhou [50] highlighted that future physicians would embody three roles: scholars and scientists, practitioners, and professionals. The American Medical College Alliance (2010) set educational goals to develop future doctors who pursue excellence in medical technology and humanistic care skills, master the scientific basis of organ systems, apply this knowledge in clinical practice, and lead team-based innovation.

#### Taxonomies of Professional Development

Fink’s [14] taxonomy presents an interactive approach with six levels: foundational knowledge, application, integration, human dimension, caring, and learning how to learn. This taxonomy emphasizes that each type of learning can catalyze other types. Foundational knowledge involves memorizing and comprehending information, while application encompasses critical, creative, and practical thinking skills. Integration refers to the integration of knowledge, ideas, perspectives, and learning experiences. The human dimension covers personal and social experience, including metacognitive skills and interactive skills with others. Caring involves developing attention and interest in new subjects, and learning how to learn which is based on self-regulation skills for continuous learning.

Krathwohl et al. [25] taxonomy describes an internalizing process where learners gradually align their actions with a set of values. This process includes five levels: receiving, responding, valuing, organization, and characterization. Learners evolve from being sensitive to ideas and phenomena to forming consistent behavior aligned with their value system. On the other hand, a bidimensional skill development model outlines two levels of competencies: intellectual skills and operational skills. Academic skills involve cognitive processes such as calculation and reading, while operational skills develop through stages: cognitive orientation, action imitation, action integration, and skilled operation (Liu et al., 2017).

### Assessment Methods

#### Evaluation Framework of Medical Education

Four levels of training evaluation include reaction, learning, behaviors, and results. Reaction assesses learners’ satisfaction and confidence, learning assesses progress through quizzes and tests, behaviors measure application in clinical settings through Objective Structured Clinical Examinations (OSCE), and results assess professional practice performance [23]. Miller’s pyramid, a widely used model, divides clinical competence into four levels: knows, knows how, shows, and does [29]. Lower levels focus on cognitive knowledge, while upper levels emphasize behavioral application. Studies indicate the complexity of transitioning from cognitive understanding to behavioral execution, highlighting the need for practical evaluation in clinical settings [47].

#### Clinical Assessments

Introduced the OSCE to evaluate medical students’ clinical practice ability, focusing on cognition, psychomotor skills, and emotion [19]. This comprehensive assessment method is widely adopted because of its high reliability. Ken Cox (1993) emphasized clinical skills training, highlighting the importance of improving students’ clinical thinking and innovation. In the 1990 s, core standards for clinical medical skills training were established, leading to the competency-based clinical training index by the American Accreditation Council for Postgraduate Medical Education [11]. This index includes six core competencies: patient diagnosis and treatment, medical knowledge, interpersonal communication, professional literacy, practice-based learning and improvement, and system-based practice [38]. The International Society of Medical Education’s “Global Minimum Essential Requirements in Medical Education” (GMER) outlined fundamental requirements for medical knowledge, skills, professional attitudes, behaviors, and values [35].

### Digitalized Medical Education

#### The Digitalized Medical Education

Tudor Car et al. [41] developed a conceptual framework for digital education in health majors comprising six core dimensions: context, infrastructure, education, learners, research, and quality improvement. The context includes sociocultural factors and educational norms, infrastructure refers to physical instruments to deliver content, and learners are central, with unique needs and competencies influenced by their context. Each dimension dynamically interacts with the others.

#### Modalities of Digitalized Medical Education

Offline education delivers content through external media without requiring internet access, suitable for regions with limited internet infrastructure [21]. Online education requires Internet access and includes multimedia formats such as video conferencing and streaming [32]. Massive open online courses (MOOCs) provide free and accessible programs to large audiences [37]. Mobile education (m-Learning) offers flexible learning through personal devices [12]. Serious games and gamification test knowledge and cognitive skills in a virtual environment [18]. Simulated learning mimics real-life scenarios, using virtual patients and high-fidelity manikins to simulate clinical settings [10], [26]. Blended education combines digital and traditional methods, integrating online and in-person interactions.

#### The Digitalized Approach Versus Traditional Approach in Medical Education

Studies that compare digital and traditional approaches show mixed results. Bhone et al. [4] found that offline digital learning is as effective as traditional methods for acquiring knowledge and is superior for skill improvement. Zhang et al. [48] demonstrated that digital simulation technology in OSCE exams improves students’ interests, motivation, communication, and operational skills. However, Tudor Car et al. [42] found no significant differences in learning outcomes between digital and traditional methods but noted greater satisfaction with digital education. Gao et al. [15] found no significant improvement in learning outcomes with digital education but noted greater satisfaction with the OSCE information system. While Zhang et al. [48] reported a 15% improvement in OSCE scores with digital simulation, Gao et al. [15] found no significant difference in performance between digital and traditional methods (*p* = 0.12), highlighting the need for context-specific implementation.

#### Medical Intern’s Digital Health Competence

The Chinese Medical Digital Health Competence Scale, adapted from Scott et al. [36] competencies for the Royal Australasian College of Physicians (RACP), assesses digital health competency in Chinese medical students. Studies show a moderate level of digital competence and a high level of digital ethics among Chinese medical students [17]. The level predicts digital literacy, but overall competence, including awareness of digital health and information management, remains low [46]. Digital literacy impacts learning motivation and outcomes [16]. Training can improve information literacy and personal abilities [44], but excessive information can cause overload [31].

### Studies of the Impact of Social-Ecological Factors on Medical Graduates’ Clinical Performance

Various factors affect the clinical operations skills of medical students. Policy, system, and management issues can contribute to performance declines. He et al. [20] highlighted the need for more clinical rotation opportunities and accurate work rotation. Lu et al. [27] identified the teacher-student ratio as a factor affecting clinical performance.

Assessment standards also impact performance. He et al. [20] called for a well-structured clinical skills assessment system, citing issues with current assessment tools and procedures. Chen and Hu [9] noted the need for standardized assessment scales and refined scoring items.

Meanwhile, cognitive and psychological factors, such as self-regulated learning and anxiety, affect clinical performance. Martin and Naziruddin [28] found a minimal impact of OSCE-related anxiety, while Arain [1] found that anxiety negatively affects academic operational skills, especially in female students. Empathy levels also impact performance [7].

Demographic factors, such as age and gender, influence operational skills. Bai [2] found that female students generally perform better than male students in clinical evaluations. Klein et al. (2019) noted that gender bias in assessments can influence results. Economic factors, such as income and debt, also affect performance [33].

Besides that, teaching approaches and instructor-student interaction significantly affect clinical skill development [7]. Found that participatory teaching methods improve performance compared to traditional methods. Zhang et al. (2022) emphasized the positive impact of instructor motivation and guidance on clinical performance.

## Discussions

This review has limitations. First, most studies relied on self-reported data, which may introduce response bias. Second, the predominance of cross-sectional designs limits causal inference.

### Research Gap

The relationship between contributing factors and the decline in operational skills among medical interns is understudied. From both global and Chinese perspectives, the existing literature provides inconsistent findings regarding factors that could explain the decline in clinical and operational skills. More importantly, there is a need to improve the rigor of the examination of the predictive relationships between these constructions. Differentiations in participants, research contexts, and methods contribute to the variation in results. Researchers have found that junior doctors’ operational skills can be influenced by a broader and more complex set of factors than just clinical competence alone [43]. Most previous studies rely on surveys, interviews, or assessments with relatively small sample sizes, which can limit the generalizability of the findings. For example, Tang et al. (2022) conducted qualitative research to investigate the negative factors that influence the operational skills of general practitioners (GP). They identified five key factors that affect practitioner trainees in two cities in China: low social recognition, low professional identity, low levels of remuneration, an imperfect training system, and the influence of policy factors. However, it is important to note that this study focused on a small subset of clinical professions within two eastern Chinese cities, which may not fully represent the broader medical field. The need for more comprehensive research is clear. Future studies should aim to include larger and more diverse samples and employ various research methodologies to provide a more robust understanding of the factors influencing the decline in operational skills. Addressing these gaps could help to develop targeted interventions and policies to improve the training and performance of medical interns, ensuring that they are better prepared for the demands of their profession.

Second, there is a pressing need for a more holistic approach to assessing the clinical operational skills of post-graduate students. Currently, there is no unified standard for evaluating clinical doctoral students in China, and there is a significant gap between the intended assessment goals and their actual implementation [20]. Although scholars have identified the Objective Structured Clinical Examination (OSCE) as the most appropriate summative assessment tool to identify deficiencies in clinical operational skills in the workplace, researchers and policymakers should approach the data from these studies with caution (Terry, 2017). Many studies have focused on specific aspects of research competence (Bai et al., 2020,Ju et al., 2020; Li et al., 2021; Liu & Ma, 2020; Zhang et al., 2019). However, there is a lack of research that adopts a more holistic conceptual framework. An exception is Bai [2], whose study encompassed a broader perspective. However, their data was obtained several years ago and focused on attending trainees in Canada and postgraduates in China. This highlights the need for further research with updated data, specifically on undergraduate students, a group that has been largely overlooked.

Undergraduate students face unique challenges and contexts that could influence their clinical operational skills differently compared to undergraduates or trainees in other countries. Therefore, understanding the factors that affect their operational skills is crucial. These factors can exhibit different patterns and levels of impact, necessitating a comprehensive approach to identify and address them effectively. Renewed research efforts should aim to develop a unified and holistic assessment framework that accurately reflects the multifaceted nature of clinical operational skills, ensuring that undergraduate students are well prepared for their professional responsibilities.

Given the existing literature, there is a notable lack of research evaluating behavior change in healthcare professionals after digital education interventions. An exception is a study by Tudor et al. (2019), which focused on spaced education and found it beneficial in improving students’ operational skills. They also discovered that interactive online games could improve patient outcomes. In contrast, Chinese researchers found that digital systems did not significantly outperform traditional paper-based methods in improving student learning outcomes [15]. However, it was observed that both the instructors and the students of the experimental group showed higher levels of satisfaction with the OSCE information system compared to the control group using non-digital OSCE methods. Furthermore, Liu et al. (2022) explored the role of big data technology in promoting Chinese medical students’ digital competencies and professionalism through a workshop experiment. The results indicated that digital training could enhance the understanding of digital health by medical students and their sense of professionalism. These findings suggest that, while digital education tools may not always directly translate into better learning outcomes compared to traditional methods, they can significantly impact user satisfaction and understanding of digital health concepts. Therefore, more research is needed to fully understand the potential of digital education in transforming healthcare education and practice.

A noticeable scarcity of research investigating the digital competence of Chinese medical students exists. Only one study has focused specifically on the relationship between digital competence and academic performance, as reported by Sun et al. (2023). Most existing studies have used surveys to collect data on medical students, which has led to conflicting results and findings. For example, Ge et al. [17] found that medical undergraduates typically exhibited a moderate level of digital competence along with a high level of digital ethics. In contrast, Wu et al. [46] study suggested that the level of education could predict medical students’ digital literacy. Despite this, statistics revealed that Chinese medical students generally have a low level of digital competence, encompassing areas such as digital health awareness, information management, and digital ethics. Pan [31] is the only researcher to adopt a grounded theory approach to explore the factors that influence the digital health literacy of medical students through structured interviews. This method provided a more in-depth understanding of the underlying factors that impact digital competence. Despite these efforts, the general impact of digital health competency on clinical performance has received little attention in the existing literature. This gap highlights the need for more comprehensive research to understand how digital skills and literacy affect medical students’ clinical abilities and professional development.

In summary, although some studies have begun to shed light on the digital competency of Chinese medical students, the findings are often inconsistent, and the research methods vary significantly. There is a pressing need for more detailed and systematic research to better understand the digital competencies required for effective clinical performance and how these skills can be cultivated through medical education. This would help bridge the current knowledge gap and contribute to improving the digital literacy and clinical skills of future medical professionals in China.

### Comparison of Various Studies

The comparison in Table 1 provides a comprehensive overview of various studies and frameworks related to medical education, specifically focusing on clinical skills, educational methods, and digital learning resources. The articles listed span a variety of topics, such as the development of clinical skills, training evaluations, digital education, and the integration of technology into medical education.

**Table 1:** Comparison of the results of the literature review

Article	G/PG	Theme	Resources	Active method		
				CSA	DME	SEF
[49]	G	Operation skills (motor skills)	R	A	N/A	N/A
[8]	G	Medical doctor roles (advocator, educator, etc.)	N/A	A	N/A	N/A
[5]	G	Five-Star Doctor (health care, communication, leadership)	R	A	N/A	N/A
[45]	G	Global standards for medical graduates	V	A	N/A	N/A
[50]	G	Future doctor roles (scholars, practitioners, professionals)	N/A	A	N/A	N/A
[AMCA, 2010]	PG	Educational goals for future doctors (medical tech, humanistic care)	R	A	N/A	N/A
[Fink, 2003]	G	Taxonomy of professional development (foundational knowledge, application)	R	A	N/A	N/A
[25]	G	Internalizing process in learning values (receiving, responding, etc.)	N/A	A	N/A	N/A
[Liu et al., 2017]	G	Bi-dimensional skill development (intellectual skills, operational skills)	N/A	A	N/A	N/A
[Kirkpatrick, 2011]	G	Training evaluation (reaction, learning, behavior, results)	V/R	A	N/A	N/A
[29]	G	Clinical competence model (knows, knows how, shows, does)	V/R	A	N/A	N/A
[19]	G	OSCE for clinical practice ability assessment	R	A	N/A	N/A
[Ken Cox, 1993]	PG	Clinical skills training (clinical thinking, innovation)	N/A	A	N/A	N/A
[11]	PG	Core competencies for clinical skills (diagnosis, treatment, communication)	R	A	N/A	N/A
[35]	G	GMER — global minimum essential requirements	N/A	A	N/A	N/A
[41]	G	Digitalized medical education framework (context, infrastructure, etc.)	R	A	A	N/A
[21]	G	Offline education	N/A	N/A	A	N/A
[32]	G	Online education (multimedia, video conferencing)	R	N/A	A	N/A
[37]	G	Massive open online courses (MOOCs)	R	N/A	A	N/A
[12]	G	m-Learning (mobile education)	R	N/A	A	N/A
[18]	G	Serious gaming and gamification	R	N/A	A	N/A
[10]	G	Simulated learning (virtual patients, high-fidelity manikins)	R	N/A	A	N/A
[26]	G	Simulated learning (clinical scenario simulation)	R	N/A	A	N/A
[4]	G	Digital learning vs traditional (effectiveness, skill improvement)	N/A	N/A	A	N/A
[48]	G	Digital simulation in OSCE (enhances interest, motivation, and skills)	R	N/A	A	N/A
[42]	G	Digital vs traditional education comparison (learning outcomes, satisfaction)	R	N/A	A	N/A
[15]	G	Digital OSCE information system (effect on satisfaction)	R	A	A	N/A
[17]	G	Digital health competence (Chinese medical students)	R	A	A	N/A
[46]	G	Education level and digital literacy prediction	N/A	N/A	A	N/A
[44]	G	Digital literacy improvement through training	R	N/A	A	N/A
[31]	G	Information overload and digital health literacy	R	A	A	N/A
[20]	G	Policy and system impact on clinical skills performance	N/A	N/A	N/A	A
[27]	G	Teacher-student ratio and clinical performance	R	A	N/A	A
[7]	G	Participatory teaching methods and clinical performance	R	A	N/A	N/A
[Zhang et al., 2022]	G	Instructor motivation and guidance impact on clinical skills	R	A	N/A	A
[Tang et al., 2022]	PG	Negative factors in practitioner trainees (low recognition, identity, etc.)	R	N/A	N/A	A
[Terry, 2017]	G	OSCE assessment and clinical skills deficiencies	N/A	A	N/A	N/A
[2]	G	Clinical skills and assessment gaps for postgraduate students	R	A	N/A	N/A
[Liu et al., 2022]	G	Big data in digital training for medical students'professionalism	R	N/A	A	N/A

*CSA* clinical skill assessment, *DME* digitalized medical education, *SEF* social-ecological factor, *PG* graduate students, *G* undergraduate students, *v* virtual, *R* real, *N/A* not applicable, *F* formative, *s* summative

Table 1 categorizes 39 studies into four themes: clinical skill training (CSA), digitalized medical education (DME), assessment methods, and social-ecological factors (SEF). Key findings indicate that 60% of studies focused on CSA, while only 18% addressed SEF, reflecting a research gap in socio-contextual influences.

Many studies emphasize the importance of operational and motor skills for medical professionals. For example, Zhao and Guo [49] focus on motor skills essential for clinical practice, while Liu et al. (2017) examine the bidimensional development of both intellectual and operational skills. Other sources, such as Kirkpatrick (2011) and Miller’s Pyramid model of clinical competence, categorize it into stages such as “knows,” “knows how,” “shows,” and “does.” The OSCE, introduced by [19], is another vital assessment tool used for evaluating clinical practice abilities. Several studies highlight the evolving roles of medical professionals, such as the “Five-Star Doctor” framework by Boelen [5], which identifies competencies such as healthcare care, communication, and leadership. The roles of future doctors, as conceptualized by Zhou [50] and the ACGME (2000), focus on developing core competencies such as diagnosis, treatment, and communication. The educational goals of future doctors, as defined by AMCA (2010), emphasize medical technology and humanistic care.

Many studies discuss frameworks for evaluating medical training and clinical skills. Miller’s pyramid and the Kirkpatrick evaluation model focus on the different levels of training outcomes, from reaction and learning to behavior and results. These frameworks are essential to understanding how clinical competencies are measured and how to improve training strategies. Meanwhile, the integration of digital tools into medical education is extensively explored. Studies such as those by Paul et al. [32] and [37] highlight online education, multimedia resources, and massive open online courses (MOOCs), showing how these digital platforms enhance learning flexibility and access. Other studies focus on specific digital resources, such as mobile learning (m-Learning) [12] and serious gaming [18], that engage students in interactive and immersive learning environments.

Several studies compare digital and traditional learning methods in terms of effectiveness and results. For example, Bhone et al. [4] and Zhang et al. [48] investigated the role of digital simulation in OSCEs and its impact on student motivation and skill development. These studies suggest that digital tools can significantly enhance both engagement and performance, while others, like Tudor Car et al. [41] and Gao et al. [15], explore how digital systems, including information systems in OSCEs, contribute to improved satisfaction among medical students. Some studies focus on the challenges faced in medical training, such as the impact of policy on clinical skills performance [20] or factors such as teacher-student ratios and participatory teaching methods [7, 27]. These studies emphasize the need for an effective educational system that balances quality and the infrastructure that supports the medical training environment.

The studies highlighted in Table 1 collectively underscore the need for continuous innovation in medical education. By integrating traditional and digital methods, there is an opportunity to improve clinical competency, improve learning outcomes, and better prepare future physicians for the dynamic demands of healthcare. Exploring various teaching methods, from hands-on clinical assessments such as OSCE to digital learning frameworks, provides a comprehensive approach to medical training in the modern era. Through a systematic review of the existing literature, the researcher found that although significant progress has been made in the integration of digital education methods and clinical skills training in medical education, there are still several key issues that have not been fully explored. First, although most studies support the effectiveness of digital education in improving students’ knowledge and skills, its performance in long-term effect evaluation and practical application is still controversial. Second, in terms of evaluation methods, existing research focuses mainly on the measurement of single indicators while ignoring the need for a comprehensive evaluation.

## Conclusions

Current research still has some important deficiencies, especially in terms of the applicability of digital education in different teaching situations and the impact of individual student differences on educational effectiveness. These shortcomings provide directions for future research, especially in the discussion of various assessment methods and interdisciplinary education models.

Future studies should prioritize longitudinal designs to assess skill retention over 5–10 years, comparing digital and traditional cohorts in real clinical settings, especially their application in actual clinical situations. Additionally, there are more diverse and comprehensive assessment tools that more accurately reflect the full capabilities of students. Through these efforts, we can not only deepen our understanding of the digital transformation of medical education but also provide more reliable tools and methods for educational practitioners. To bridge the gap between research and practice, researchers recommend piloting blended learning programs in Chinese medical colleges, combining virtual simulations with supervised clinical rotations.

In summary, this review clarifies the main findings, limitations, and potential directions of future research in existing research, indicating the urgency and necessity of further exploring the integration of digital education and traditional education in medical education. This analysis provides a solid theoretical and practical foundation for future research and is expected to promote the development of this field.

## Supplementary Information

Below is the link to the electronic supplementary material.

Supplementary file1 (DOCX 24 KB)

## Data Availability

The datasets used and/or analyzed during the current study are not publicly available due to institutional confidentiality and sensitivity of the data. However, they are available from the corresponding author on reasonable request.
