# Supplementary material for: Deviation Factors of Medical Operational Skills in Terms of Clinical Skills Training, Assessment Methods, and Digitalized Education — A Prisma-Based Review
Source: Med Sci Educ. 2025 Jul 19;35(4):2237–47. doi: 10.1007/s40670-025-02407-7 (PMC12532553; doi:10.1007/s40670-025-02407-7)
Supplement: Supplementary file 1 — Supplementary file1 (DOCX 24 KB) [file 40670_2025_2407_MOESM1_ESM.docx]

**Supplementary Information**

Supplementary File 1

The following search strings were used across databases to identify relevant studies for the systematic review. Boolean operators (AND/OR), truncation symbols (*), and database-specific syntax were applied to optimize retrieval.

1. PubMed

Search Date: [Insert Date]

Filters: 2018–2023, English/Chinese

("medical education"[MeSH Terms] OR "medical training" OR "clinical education")

AND

("clinical skills"[MeSH Terms] OR "clinical competence" OR "operational skills" OR "motor skills")

AND

("digital education"[tiab] OR "digitalized education"[tiab] OR "e-learning"[tiab] OR "online learning"[tiab] OR "virtual simulation"[tiab] OR "serious gaming"[tiab])

AND

("assessment methods"[tiab] OR "OSCE"[tiab] OR "Miller's pyramid"[tiab] OR "Kirkpatrick model"[tiab] OR "clinical evaluation"[tiab])

NOT

("animal studies"[tiab] OR "case report"[ptyp])

2. Web of Science

Search Date: [Insert Date]

Filters: 2018–2023, English/Chinese

TS= ("medical education" OR "clinical training")

AND

TS= ("clinical skills" OR "operational skills" OR "clinical competence")

AND

TS= ("digital education" OR "digitalized education" OR "e-learning" OR "online learning" OR "virtual simulation")

AND

TS= ("assessment methods" OR "OSCE" OR "clinical evaluation" OR "Kirkpatrick framework")

NOT

TS= ("animal" OR "pediatric" OR "veterinary")

3. Google Scholar

Search Date: [Insert Date]

Filters: 2018–2023, English/Chinese

allintitle: ("medical education" OR "clinical training")

AND

("clinical skills" OR "operational skills")

AND

("digital education" OR "online learning" OR "virtual simulation")

AND

("assessment methods" OR "OSCE" OR "Miller's pyramid")

-filetype:pdf -patent

4. CNKI (Chinese Database)

Search Date: [Insert Date]

Filters: 2018–2023

SU=('医学教育' OR '临床培训')

AND

SU=('临床技能' OR '操作技能')

AND

SU=('数字化教育' OR '在线学习' OR '虚拟仿真')

AND

SU=('评估方法' OR '客观结构化临床考试' OR 'OSCE')

5. Scopus

Search Date: [Insert Date]

Filters: 2018–2023, English/Chinese

TITLE-ABS-KEY (("medical education" OR "clinical training")

AND

TITLE-ABS-KEY (("clinical skills" OR "operational skills"))

AND

TITLE-ABS-KEY (("digital education" OR "online learning" OR "virtual simulation"))

AND

TITLE-ABS-KEY (("assessment methods" OR "OSCE" OR "clinical evaluation"))

NOT

TITLE-ABS-KEY ("animal" OR "pediatric")

6. Explanation of Search Strategy

6.1. Core Themes: Keywords were grouped into four clusters aligned with the review’s objectives:

Clinical Skills Training: "clinical skills," "operational skills," "motor skills."

Digitalized Education: "digital education," "e-learning," "virtual simulation."

Assessment Methods: "OSCE," "Miller's pyramid," "Kirkpatrick model."

Exclusion Terms: Animal studies, pediatric/veterinary contexts.

6.2. Syntax Adjustments:

Truncation (*) captured variations (e.g., "simulat*" for "simulation/simulate").

Boolean operators (AND/OR) linked related terms while narrowing scope.* *

Database-specific syntax (e.g., `[MeSH Terms]` in PubMed, `SU=` in CNKI) ensured precision.

6.3. Language and Date Filters: Limited to English and Chinese studies published between 2018–2023 to prioritize recent evidence.
